# Pemetrexed-based first-line chemotherapy had particularly prominent objective response rate for advanced NSCLC: A network meta-analysis

**DOI:** 10.1515/med-2021-0202

**Published:** 2021-01-27

**Authors:** Yuankai Lv, Zhuo Cao, Jiongwei Pan, Enhui Gong, Hao Zheng, Xiaoping Cai

**Affiliations:** Department of Respiratory, Lishui People’s Hospital, No.15 Dazhong Road, Liandu District, Lishui, Zhejiang, 323000, People’s Republic of China

**Keywords:** non-small cell lung cancer, first-line chemotherapy, network meta-analysis, objective response rate

## Abstract

**Objective:**

The aim of the present work was to investigate the clinical efficacy of first-line chemotherapy regimens in the treatment of advanced non-small cell lung cancer (NSCLC) through a comprehensive network meta-analysis (NMA).

**Methods:**

The prospective randomized controlled clinical trials relevant to 10 first-line chemotherapy regimens in the treatment of advanced NSCLC were systematic electronic search in the databases of Pubmed, Embase, Cochrane Library and CNKI. The combined direct or indirect objective response rate (ORR) between each of the 10 first-line chemotherapy regimens was calculated.

**Results:**

Seventeen prospective clinical trials of first-line chemotherapy regimens in treatment of advanced NSCLC were included in the NMA. The 10 treatment regimens including A = cisplatin + gemcitabine, B = carboplatin + gemcitabine, C = gemcitabine, D = carboplatin + paclitaxel, E = paclitaxel + gemcitabine, F = docetaxel + carboplatin, G = gemcitabine + vinorelbine, H = pemetrexed + carboplatin, I = cisplatin + pemetrexed and J = cisplatin + docetaxel were compared in the present NMA. Direct pooled results indicated that the ORR was not statistically different (*P*_all_ > 0.05). However, NMA showed that the combined ORR for regimens A (OR = 1.47, 95% CI: 0.80–2.81), B (OR = 3.22, 95% CI: 1.45–6.923), D (OR = 3.30, 95% CI: 1.22–9.33), E (OR = 4.36, 95% CI: 1.64–12.82), G (OR = 3.72, 95% CI: 1.12–12.83) and I (OR = 5.80, 95% CI: 2.04–17.86) was superior to regimen C. Rank probability analysis indicated that regimen C = gemcitabine and regimen I = cisplatin + pemetrexed had the highest probability of inferior and superior treatment ORR among the 10 first-line chemotherapy regimens.

**Conclusion:**

Cisplatin + pemetrexed may have particularly prominent ORR for advanced NSCLC as the first-line chemotherapy regimen.

## 1 Introduction

Lung cancer is known as the leading cause of cancer-relevant death worldwide and the most diagnosed carcinoma among male and the second most among female [1]. The morbidity of lung cancer in China is about 530/100,000 according to the recent tumor epidemiology data [2]. Lung cancer accounts for 18.74% of the newly diagnosed cancer cases annually [3], and 80% of the newly diagnosed lung cancer cases are at advanced stages with a poor 5-year survival rate of lower than 15% [4]. Lung cancer is generally divided into non-small cell lung cancer (NSCLC) and small cell lung cancer according to the pathology findings. NSCLC accounts for 80% of all the diagnosed lung cancer. However, most of the NSCLC are at advanced or locally advanced stages, would have lost the opportunity for surgery, with the poor overall survival. The standard treatment of the patients with newly diagnosed advanced NSCLC was platinum-based first-line chemotherapy regimens including cisplatin + gemcitabine, carboplatin + gemcitabine, carboplatin + docetaxel, carboplatin + pemetrexed, cisplatin + docetaxel, etc. Compared with the best supportive care, chemotherapy can improve the 1-year survival rate by 9% [5]. A number of clinical trials from Southwest Oncology Group (SWOG) and Eastern Cooperative Oncology Group (ECOG) showed that the response rate for DDP/CBP + PTX/VNR/DXI/GEM were not significantly different without considering the histological type for advanced NSCLC [6,7]. A meta-analysis found that compared with other platinum-based two-drug regimen, platinum + GEM can reduce the risk of death by 10% [8]. The response rate of first-line chemotherapy regimen for advanced NSCLC was not significantly different as shown by early clinical trials that did not further discriminate the pathological types of NSCLC. The response and adverse reactions of different platinum-based dual-drug regimens in patients with advanced NSCLC were similar.

Several meta-analyses have directly compared the response of platinum-based chemotherapy regimens, and most of them found negative results for advanced NSCLC [9,10]. However, not all the chemotherapy regimens were directly compared by prospective randomized controlled trials or meta-analysis. The response of these chemotherapy regimens without direct comparison was unclear. Therefore, we performed this network meta-analysis (NMA) in order to further evaluate the clinical efficacy of the first-line chemotherapy for advanced NSCLC.

## 2 Methods

### 2.1 Screening of electronic databases of Studies

Clinical trials about the first-line chemotherapy regimens in the treatment of advanced NSCLC involved systematic search of the databases of Pubmed, Embase, Cochrane Library and CNKI. The electronic search words were “non-small cell lung cancer,” “lung squamous cell carcinoma,” “squamous cell carcinoma of the lung,” “lung adenocarcinoma,” “adenocarcinoma of the lung,” “chemotherapy” and “first-line.” The clinical trials that were identified initially from the relevant databases were further independently screened by Yuankai Lv and Zhuo Cao and reviewed by Xiaoping Cai. The references of the included studies were also screened in order to identify the potential and suitable clinical trials.

### 2.2 Inclusion of studies and data extraction

The studies were further screened to identify the suitable clinical trials for data pooling. The studies should fulfill the below items for inclusion. (1) The study type should be prospective randomized clinical trials. (2) The patients of the original studies should be pathology- or cytology-confirmed NSCLC in advanced stages. (3) The patients of the original studies should have received the first-line chemotherapy regimens. (4) The objective response ratio (ORR) requires to be extracted or calculated from the original study. (5) Studies published in either English or Chinese. The studies exclusion criteria include (1) retrospective study, case report or review; (2) small cell lung cancer patients or patients without pathology or cytology confirmation, (3) neoadjuvant chemotherapy for early stage NSCLC and (4) duplicated publications or data.

### 2.3 Quality assessment

The general methodical quality of the included clinical trials were independently assessed by two reviewers (Jiongwei Pan and Enhui Gong) and checked by Xiaoping Cai. The general quality of the included studies was evaluated by a six-item questionnaire including adequate sequence generation? Allocation concealment? Blinding? Incomplete outcome data address? Free of selective reporting? Free of other bias aspects according to the Cochrane Library handbook for systematic review.

### 2.4 Statistical analysis

Stata16.0 and R3.6.2 statistical software were applied for data analysis. Network of the chemotherapy regimen interaction was performed by stata16.0. The NMA was done based on the Bayesian approach. NMA was pooled by consistency or inconsistency model according to the inconsistency test. Two-tail *p* < 0.05 was deemed statistically different.

## 3 Results

### 3.1 Publication searching

**Figure 1:**
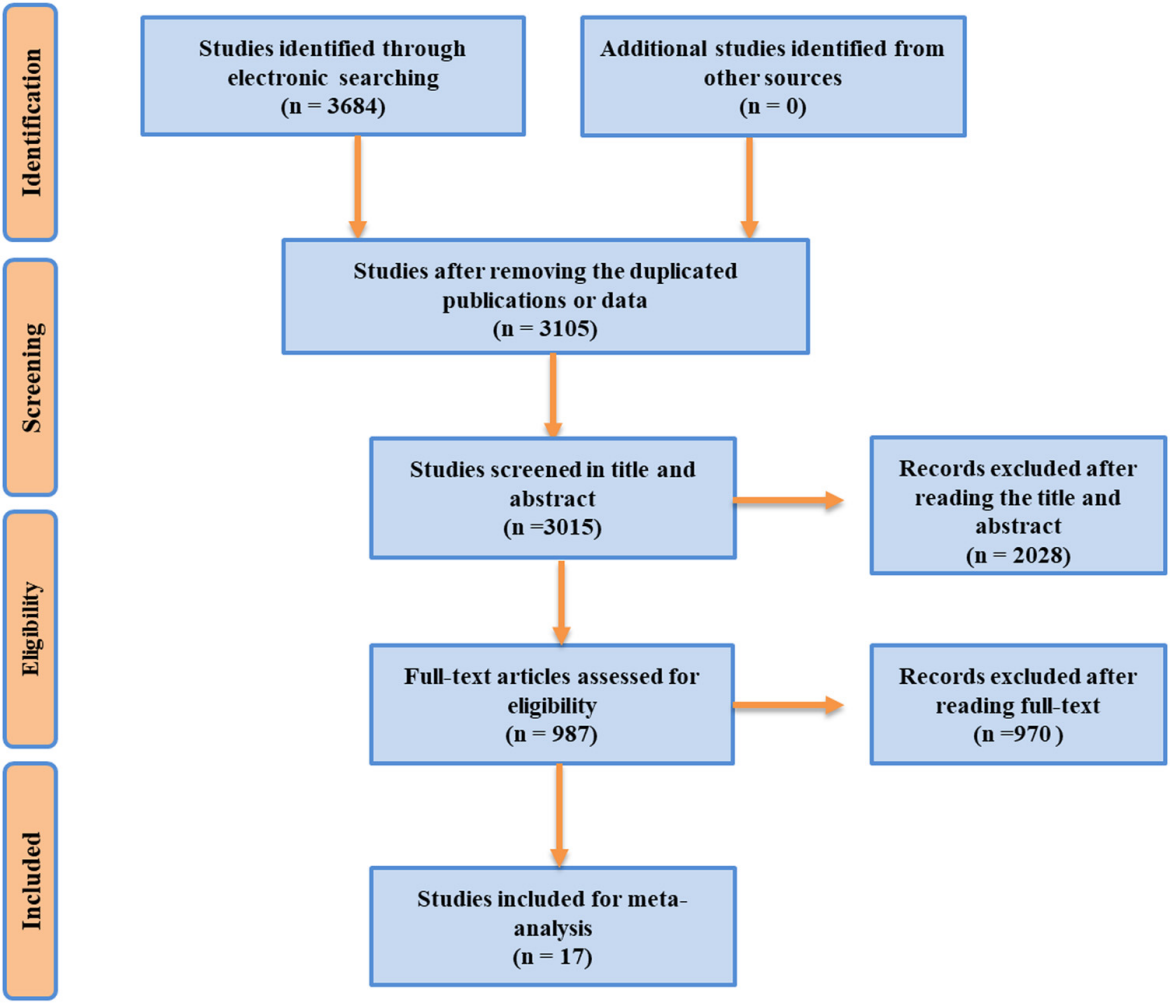
The clinical trials’ electronic search flowchart.

Through searching the relevant electronic databases of Pubmed, Embase, Cochrane Library and CNKI, 3,684 studies relevant to the first-line chemotherapy regimens in treatment of advanced NSCLC were identified. After removal of unsuitable studies, 17 randomized controlled trials were included in the NMA (Figure 1). The main features of the included clinical trials are shown in Table 1.

**Table 1:** General characteristics of the included studies

Study	Year	Region	Treatment		Sample size		Response	
			T1	T2	n1	n2	R1	R2
Yang et al. [11]	2016	China	H	B	84	60	44	32
Wu et al. [12]	2014	China	A	I	116	121	24	30
Xing et al. [13]	2014	China	I	G	49	49	22	20
Minami et al. [14]	2013	Japan	B	D	25	25	4	9
Kusagaya et al. [15]	2012	Japan	B	C	31	30	7	3
Huang et al. [16]	2012	China	I	A	127	127	31	18
Biesma	2011	The Netherlands	B	D	74	68	20	13
Xie et al. [17]	2011	China	J	I	21	19	5	6
Socinski et al. [18]	2010	U.S.	H	F	74	72	16	20
Vergnenegre et al. [19]	2009	France	A	E	45	42	7	9
Kosmidis et al. [20]	2008	Greece	B	E	227	225	62	70
Scagliotti et al. [21]	2008	Italy	I	A	839	830	257	234
Langer et al. [22]	2007	America	A	D	100	100	23	14
Yi et al. [23]	2006	China	J	F	21	24	9	9
Sederholm et al. [24]	2005	Sweden	B	C	142	59	42	18
Lilenbaum et al. [25]	2005	America	D	G	83	82	14	12
Zatloukal et al. [26]	2003	Czech Republic	A	B	87	89	36	26

T = treatment; A = cisplatin + gemcitabine; B = carboplatin + gemcitabine; C = gemcitabine; D = carboplatin + paclitaxel; E = paclitaxel + gemcitabine; F = carboplatin + docetaxel; G = gemcitabine + vinorelbine; H = carboplatin + pemetrexed; I = cisplatin + pemetrexed; J = cisplatin + docetaxel.

### 3.2 Quality assessment

**Figure 2:**
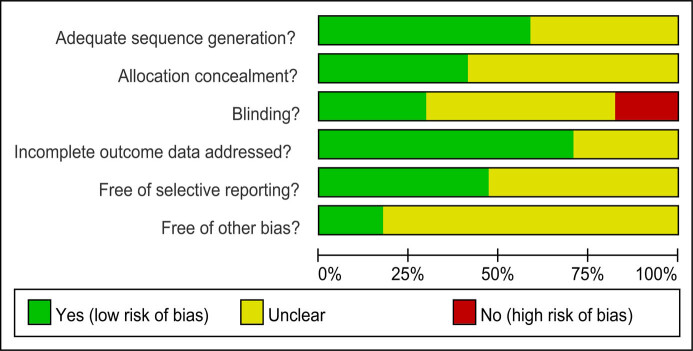
The general assessment of the included 17 clinical trials.

The methodical quality of the included 17 clinical trials is shown in Figure 2. Most of the studies had moderate risk of bias in the aspects of adequate sequence generation, allocation concealment, incomplete outcome data address and free of selective reporting.

### 3.3 Meta-analysis of direct comparison between treatments

**Figure 3:**
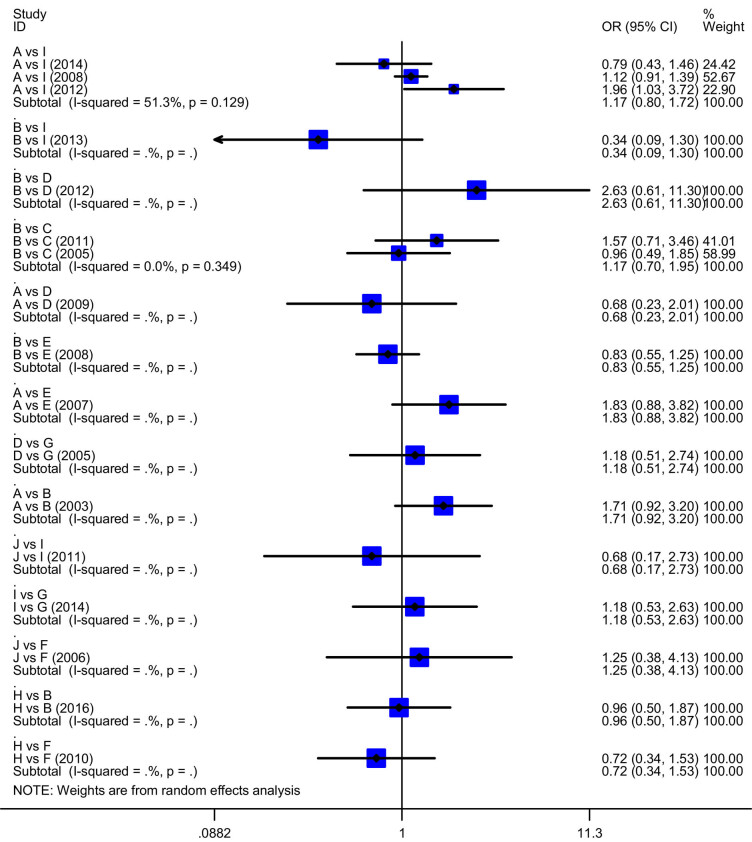
The forest plot of ORR for the first-line chemotherapy regimens in treatment of advanced NSCLC. A = cisplatin + gemcitabine, B = carboplatin + gemcitabine, C = gemcitabine, D = carboplatin + paclitaxel, E = paclitaxel + gemcitabine, F = carboplatin + docetaxel, G = gemcitabine + vinorelbine, H = carboplatin + pemetrexed, I = cisplatin + pemetrexed and J = cisplatin + docetaxel.

Ten treatment regimens included A = cisplatin + gemcitabine, B = carboplatin + gemcitabine, C = gemcitabine, D = carboplatin + paclitaxel, E = paclitaxel + gemcitabine, F = docetaxel + carboplatin, G = gemcitabine + vinorelbine, H = pemetrexed + carboplatin, I = cisplatin + pemetrexed and J = cisplatin + docetaxel. Direct pooled results indicated that the ORR was not statistically different (*P*_all_ > 0.05; Figure 3).

### 3.4 Network evidence of first-line chemotherapy regimens to treat advanced NSCLC

**Figure 4:**
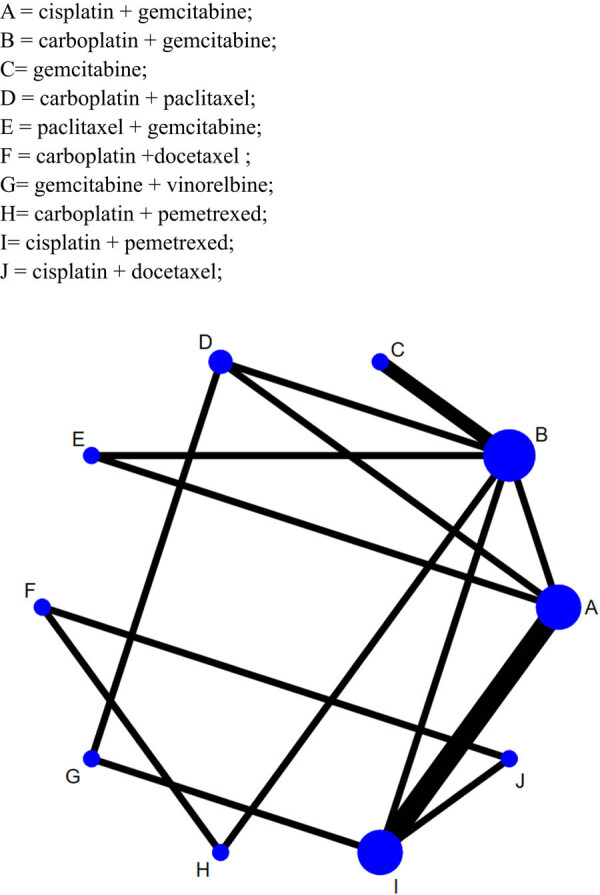
The chemotherapy regimens’ interaction network of ORR for the 10 chemotherapy regimens (Every node represented one chemotherapy regimen; the node size represented the sample size of the corresponding chemotherapy regimen; the line thickness between two nodes represented the number of RCT of two chemotherapy regimens.). A = cisplatin + gemcitabine, B = carboplatin + gemcitabine, C = gemcitabine, D = carboplatin + paclitaxel, E = paclitaxel + gemcitabine, F = carboplatin + docetaxel, G = gemcitabine + vinorelbine, H = carboplatin + pemetrexed, I = cisplatin + pemetrexed and J = cisplatin + docetaxel.

The chemotherapy regimens’ interaction network showed that the NSLCS patients mostly received regimens B = carboplatin + gemcitabine, A = cisplatin + gemcitabine and I = cisplatin + pemetrexed. Other chemotherapy regimens were rarely applied (Figure 4).

### 3.5 Inconsistency test

Node-splitting method was applied in the aspect of inconsistency analysis for the ORR among the 10 first-line chemotherapy regimens. The results indicated that direct and indirect ORRs were consistent (*P*_all_ > 0.05; Table 2). The NMA was conducted through consistency model.

**Table 2:** Inconsistency test of direct and indirect pairwise comparisons of six treatment modalities under six end point outcomes

Name	Direct effect	Indirect effect	Overall	*P* value
A, B	−0.54 (−1.53, 0.47)	−0.23 (−1.18, 0.68)	−0.38 (−1.03, 0.22)	0.61
A, D	−0.58 (−1.62, 0.39)	−0.08 (−0.99, 0.88)	−0.37 (−1.02, 0.33)	0.44
A, E	0.44 (−0.88, 1.79)	−0.30 (−1.46, 0.76)	−0.08 (−0.85, 0.75)	0.34
A, I	0.23 (−0.21, 0.83)	−0.22 (−1.67, 1.27)	0.20 (−0.21, 0.73)	0.55
B, D	0.05 (−0.80, 0.99)	−0.02 (−1.20, 1.25)	0.02 (−0.60, 0.74)	0.94
B, E	0.15 (−0.66, 1.01)	0.99 (−0.50, 2.51)	0.30 (−0.31, 1.08)	0.32
B, H	−0.04 (−1.01, 0.90)	−0.42 (−2.82, 2.08)	−0.15 (−0.99, 0.81)	0.73
D, G	−0.17 (−1.31, 0.90)	0.57 (−0.76, 1.96)	0.13 (−0.71, 0.95)	0.33
F, H	−0.26 (−1.32, 0.62)	0.51 (−2.40, 2.44)	−0.27 (−1.25, 0.67)	0.57
F, J	0.28 (−1.14, 1.56)	0.01 (−2.46, 2.18)	0.16 (−0.99, 1.37)	0.89
G, I	0.19 (−0.91, 1.25)	0.95 (−0.50, 2.32)	0.48 (−0.37, 1.30)	0.39
I, J	−0.47 (−2.05, 1.19)	−0.15 (−2.22, 2.10)	−0.28 (−1.59, 0.95)	0.73

A = cisplatin + gemcitabine; B = carboplatin + gemcitabine; C = gemcitabine; D = carboplatin + paclitaxel; E = paclitaxel + gemcitabine; F = carboplatin + docetaxel; G = gemcitabine + vinorelbine; H = carboplatin + pemetrexed; I = cisplatin + pemetrexed; J = cisplatin + docetaxel.

### 3.6 NMA

**Figure 5:**
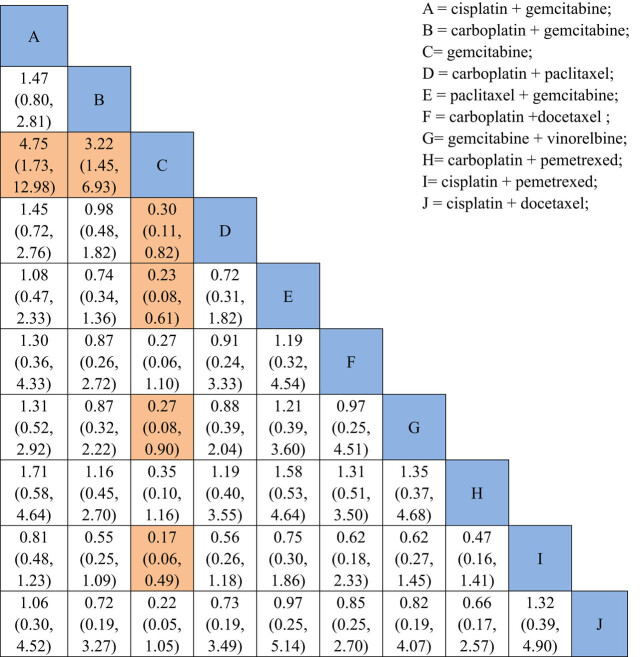
Network meta-analysis results in comparing the 10 first-line chemotherapy regimens. A = cisplatin + gemcitabine, B = carboplatin + gemcitabine, C = gemcitabine, D = carboplatin + paclitaxel, E = paclitaxel + gemcitabine, F = carboplatin + docetaxel, G = gemcitabine + vinorelbine, H = carboplatin + pemetrexed, I = cisplatin + pemetrexed and J = cisplatin + docetaxel.

NMA showed that the combined ORR for regimens A (OR = 1.47, 95% CI: 0.80–2.81), B (OR = 3.22, 95% CI: 1.45–6.923), D (OR = 3.30, 95% CI: 1.22–9.33), E (OR = 4.36, 95% CI: 1.64–12.82), G (OR = 3.72, 95% CI: 1.12–12.83) and I (OR = 5.80, 95% CI: 2.04–17.86) were superior to regimen C. Rank probability analysis indicated that regimen C = gemcitabine and regimen I = cisplatin + pemetrexed had the highest probability of inferior and superior treatment ORR among the 10 first-line chemotherapy regimens (Figure 5).

### 3.7 Rank probability

**Figure 6:**
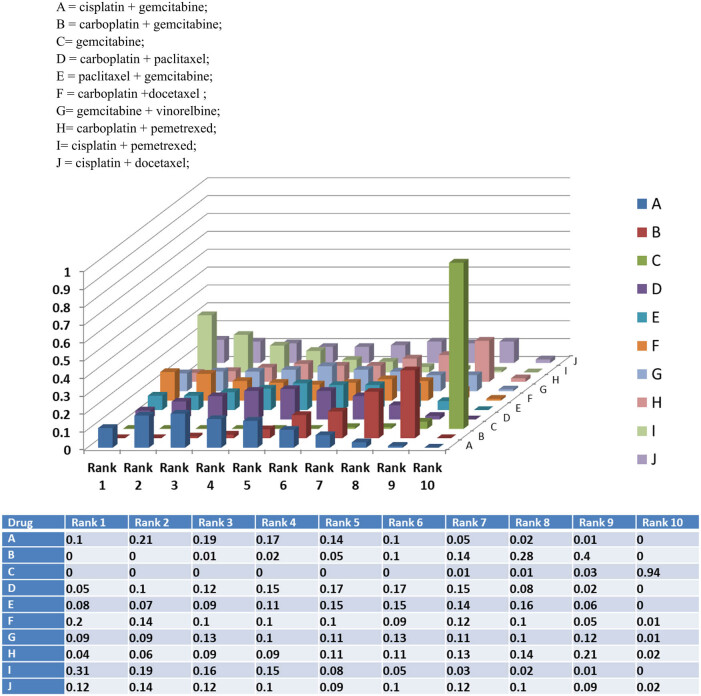
Bar plot of the rank probability of the 10 first-line chemotherapy regimens for advance NSCLC. A = cisplatin + gemcitabine, B = carboplatin + gemcitabine, C = gemcitabine, D = carboplatin + paclitaxel, E = paclitaxel + gemcitabine, F = carboplatin + docetaxel, G = gemcitabine + vinorelbine, H = carboplatin + pemetrexed, I = cisplatin + pemetrexed and J = cisplatin + docetaxel.

Rank probability analysis indicated that regimen C = gemcitabine and regimen I = cisplatin + pemetrexed had the highest probability of inferior and superior treatment ORR among the 10 first-line chemotherapy regimens (Figure 6).

## 4 Discussion

Although new chemotherapy drugs have been developed and used in clinic, the prognosis of lung cancer is still poor for advanced stage cases. For patients with advanced NSCLC of relative well-performance status, standardized chemoradiation treatment should be applied as early as possible, which can prolong the overall survival and improve the patients’ life quality [27]. Three generation chemotherapy drugs (gemcitabine, paclitaxel, docetaxel, irinotecan and vinorelbine) are the standard first-line treatment of advanced NSCLC [28]. Studies have shown that gemcitabine-based chemotherapy had a higher disease control rate, while paclitaxel-based chemotherapy has a higher risk of progression [29].

Several meta-analysis [9,10] have directly compared the response of platinum-based chemotherapy regimens and most of them found negative results for advanced NSCLC. The response and long-term prognosis of the above regimens are similar. The main differences among the schemes are toxicity, drug pretreatment and treatment costs.

However, not all the chemotherapy regimens were directly compared by prospective randomized controlled trials or meta-analysis. The response of these chemotherapy regimens without direct comparison was unclear. Therefore, we performed this NMA in order to further evaluate the clinical efficacy of the first-line chemotherapy for advanced NSCLC. In our NMA, we compared the objective response of 10 chemotherapy regimen (A = cisplatin + gemcitabine, B = carboplatin + gemcitabine, C = gemcitabine, D = carboplatin + paclitaxel, E = paclitaxel + gemcitabine, F = carboplatin + docetaxel, G = gemcitabine + vinorelbine, H = carboplatin + pemetrexed, I = cisplatin + pemetrexed and J = cisplatin + docetaxel) by directly or indirectly pooling the open published data. The combined results indicated that the direct pooled ORR was not statistically different (*P*_all_ > 0.05) for all the abovementioned chemotherapy regimens. However, NMA showed that the combined ORR for regimens A (cisplatin + gemcitabine), B (carboplatin + gemcitabine), D (carboplatin + paclitaxel), E (paclitaxel + gemcitabine), G (gemcitabine + vinorelbine) and I (cisplatin + docetaxel) were superior to regimen C = gemcitabine. Rank probability analysis indicated that regimen C = gemcitabine and regimen I = cisplatin + pemetrexed had the highest probability of inferior and superior treatment ORR among the 10 first-line chemotherapy regimens. The results indicated that cisplatin + pemetrexed may have particularly prominent ORR for advanced NSCLC as the first-line chemotherapy regimen.

Pemetrexed is a multitarget antimetabolic chemotherapeutic drug [30]. Scagliotti et al. [21] first reported the efficacy of cisplatin + pemetrexed compared with cisplatin + gemcitabine regimen in the first-line treatment of advanced NSCLC. The results showed that cisplatin + pemetrexed regimen was more effective than cisplatin + gemcitabine in the aspect of overall survival for non-squamous cell carcinoma. However, for squamous cell carcinoma, no statistical difference was observed for the two chemotherapy regimens. Therefore, based on Scagliotti’s prospective clinical trial and our NMA, cisplatin + pemetrexed was recommend for NSCLC especially in patients with non-squamous cell carcinoma.

The present meta-analysis also had limitations. The statistical heterogeneity was significant; therefore, the random effect model was applied. Ten chemotherapy regimens were included in the meta-analysis, and some of the regimens only have limited original studies. The statistical power of combining this work was limited. Only studies published in English and Chinese had been searched and included in the present work. This language restriction inevitably leads to missing out on potential suitable studies.
